# Provider perceptions of an antimicrobial stewardship program in immunocompromised patients at a cancer center

**DOI:** 10.1017/ash.2026.10756

**Published:** 2026-07-03

**Authors:** Raneem H. Pallotta, Julia E. Szymczak, Emily S. Spivak, Hannah Imlay

**Affiliations:** 1 Department of Pharmacy, University of Utah Health, Salt Lake City, UT, United States; 2 Division of Epidemiology, Department of Internal Medicine, University of Utah School of Medicine, Salt Lake City, UT, United States; 3 Division of Infectious Diseases, University of Utah School of Medicine, Salt Lake City, UT, United States

## Abstract

Although provider opposition has been identified as a barrier to initiating antimicrobial stewardship programs (ASPs) among immunocompromised (IC) patients, little is known about perceptions of IC ASPs postimplementation. We assessed provider perceptions of our cancer hospital-focused ASP. Respondents found the ASP to be acceptable and value-added.

## Introduction

Antimicrobial stewardship programs (ASPs) are required by regulatory organizations due to positive impact on patient safety and clinical outcomes.^1^ However, while ASPs are commonly active in the general population, surveys have reported reluctance to implementing ASPs among immunocompromised (IC) populations because of provider opposition, partly driven by complexity of patients and evidence base gaps.^2,3^

For example, A 2016 survey of solid organ transplant (SOT) and hematopoietic cell transplant (HCT) centers identified that 69% of centers viewed provider opposition as a challenge to stewardship activities,^4^ along with diagnostic uncertainty and poorly defined evidence base for durations. A 2019 survey of ID and non-ID physicians within the American Society of Transplantation identified that 14% of ID respondents and 22% of non-ID respondents considered their patients to be too complex for antimicrobial stewardship activities; however, also noted that ASPs including team members at bedside rounds were preferred (42%) over feedback with peer comparison (35%) or restriction policies (33%).^5^

In contrast to surveys that report provider opposition to IC ASPs, several studies highlight the impact of ASPs on prescribing among SOT patients or in cancer centers^6–10^ with some also noting provider approval of their ASP implementation.^9,10^ Few surveys have examined provider perceptions of ASPs in IC hosts in settings with an active ASP. We implemented an IC ASP in our cancer hospital in 2021; here we examined our providers’ perceptions of ASP activities.

## Methods

Huntsman Cancer Institute (HCI) is a 148-bed academic National Cancer Institute-designated cancer center at the University of Utah. The IC ASP program is a physician-and pharmacist co-led program, supported by a 0.3 full-time equivalent (FTE) physician specialized in transplant ID and a 1.0 FTE ID-trained pharmacist. Our ASP implemented an in-person prospective audit and feedback (PAF) program for our oncology services in 2023 and for our hematology and HCT/cell therapy services (referred to as “BMT service”) in 2024. PAF is performed two days per week, depending on feasibility and scheduling constraints (eg, vacation and service responsibilities). We review all patients without an ID consult who are receiving treatment antimicrobials to clarify whether antimicrobials are necessary, identify opportunities to improve antimicrobial choice, duration, dosing, and route, and assess whether the patient would benefit from ID consultation. Stewardship recommendations are communicated directly to the primary teams in person. In addition to PAF, since 2021 our ASP has developed institutional guidelines, performed daily blood culture reviews, and our health system ASP has maintained a pager for questions and restricted antimicrobial approvals (available 7 days per week from 8 AM to 10 PM).

A 20-question anonymous survey was developed using REDCap and distributed via email to attending physicians, advanced practice clinicians (APCs), and pharmacists within the hematology, BMT, and medical oncology teams^11,12^ to assess acceptability and feasibility of our ASP, with a focus on our PAF program. The survey remained open for nine weeks (November 2025–January 2026), with three reminder emails sent. The survey mostly assessed responses on a Likert scale and included the validated Acceptability of Intervention Measure (AIM), as well as two free text questions (Supplemental methods). The free text responses were analyzed using thematic content analysis through group review and consensus.

## Results

### Respondents

The survey was emailed to 119 individuals, of whom 66 (55%) responded. Among respondents, 25 (38.5%) were pharmacists, 23 (35.4%) attending physicians, and 17 (26.2%) APCs (Supplemental Table 1). Respondents represented multiple services, including hematology (50.8%), medical oncology (43.1%), and BMT (29.2%), with some individuals serving on more than one service. Most respondents had been working at HCI for 1–5 years (58.5%). Mean total (SD; range) for acceptability were AIM,18.5 (2.5; 12–20), indicating high acceptability.

### Closed-Ended Questions

Responses to close-ended items reflected highly favorable perceptions of the ASP team and PAF (Table 1). Nearly all respondents (98.5%) agreed the ASP is respectful and collaborative. Regarding agreement with ASP recommendations, 27.4% reported always agreeing, 67.7% often agreeing, and 4.8% sometimes agreeing. Additionally, 12.9% of respondents reported their teams always followed ASP recommendations during rounds, 79% often followed them, 6.5% sometimes followed them, and 1.6% rarely followed them.

**Table 1.** Survey questions and responses (n = 65)

Survey item	Strongly agree, no. (%)	Agree, no. (%)	Neutral, no. (%)	Disagree, no. (%)	Strongly disagree, no. (%)
The stewardship team is respectful and collaborative.	55 (84.6)	9 (13.8)	1 (1.5)	0 (0)	0 (0)
It is easy to contact the stewardship pharmacist or physician when needed.	42 (64.6)	14 (21.5)	9 (13.8)	0 (0)	0 (0)
The stewardship team provides knowledge and education that helps improve my antibiotic use for future patients.	56 (86.2)	9 (13.8)	0 (0)	0 (0)	0 (0)
The stewardship team improves my clinical decision-making.	52 (80)	10 (15.4)	3 (4.6)	0 (0)	0 (0)
I trust the judgment of the stewardship team.	54 (83.1)	10 (15.4)	1 (1.5)	0 (0)	0 (0)
The stewardship team helps facilitate appropriate use of antimicrobials.^a^	54 (84.4)	9 (14.1)	1 (1.6)	0 (0)	0 (0)
The stewardship team respects my clinical judgment.	48 (73.8)	14 (21.5)	2 (3.1)	0 (0)	1 (1.5)
The stewardship team OR stewardship rounds interferes with my clinical decision making.^a^	4 (6.3)	2 (3.1)	2 (3.1)	26 (40.6)	30 (46.9)
The stewardship team OR stewardship improves my efficiency at work.^a^	28 (43.8)	18 (28.8)	13 (20.3)	5 (7.8)	0 (0)
I would support the continuation of in-person stewardship rounds on our service.	53 (81.5)	10 (15.4)	2 (3.1)	0 (0)	0 (0)
The antimicrobial stewardship rounds meet my approval.	47 (72.3)	10 (15.4)	8 (12.3)	0 (0)	0 (0)
Antimicrobial stewardship rounds are appealing to me.	45 (69.2)	14 (21.5)	5 (7.7)	1 (1.5)	0 (0)
I like antimicrobial stewardship rounds.	46 (70.8)	12 (18.5)	7 (10.8)	0 (0)	0 (0)
I welcome antimicrobial stewardship rounds.	48 (73.8)	13 (20)	4 (6.2)	0 (0)	0 (0)

a One missing response for this question (total n = 64 respondents).

### Open-Ended Questions

Respondents were asked to identify helpful aspects of the ASP and areas for improvement, 47 provided comments ranging in length from a single sentence to a full paragraph. Key domains of value identified by respondents included access to expertise, guidance about when to formally consult ID, support for decision making in a high-acuity and high-volume setting, educational value of rounds and access to updated evidence (Table 2). The main area for improvement identified related to desire for increased access to rounds.

**Table 2.** Notable and recurrent themes from survey responses (n = 47)

Theme	Comments exhibiting theme (%)	Illustrative quotation
Access to ID expertise	31 (65.9)	“The ability to speak as a team candidly about the overall clinical picture and hear from experts on antimicrobial stewardship” “I think they are incredibly helpful mostly from a convenience standpoint. I feel like stewardship always has our backs and is checking on our patients to help out, anticipating problems before they arise. Then rounding, they are always there to check in and give their opinions but also are open to small sidebars as needed.”
Educational value and access updated evidence	12 (25.5)	“…Additionally, it seems like they have more time to explain the reason why and can educate us on their recommendations.” “Having clinical support for decisions, providing literature. Recommendations are always the most up to date and evidence-based. It is really helpful when, as a pharmacist, I provide a recommendation which is supported by the ASP service.”
Extra oversight for decision making in a high-acuity and high-volume setting	11 (23.4)	“I feel supported by having an extra set of eyes on my patients charts. They are super sick and the service is busy. Antibiotics sometimes get forgotten past their usefulness.” “It has been helpful to review antimicrobials that can sometimes get lost in the high census of patients and what is absolutely necessary or potentially a better option”
Giving providers confidence to make stewardship-concordant decisions when they are unsure	5 (10.6)	“Giving additional confidence to discontinue antibiotics on “soft calls” or for inappropriate indications. Improving on setting appropriate durations of therapy for “grey-area” indications.”
Guidance on when to formally consult ID or guidance when the question doesn’t rise to the level of an ID consult	8 (17)	“I like knowing when it’s appropriate to get a formal ID consult.” “…so many of our patients don’t need full ID consult but could use a little side consult. Or even just a sounding board when trying to decide between things.”

## Discussion

Our single-center survey of provider perceptions to an ASP deployed at a cancer center demonstrated overall positive responses, suggesting that IC alone should not be a barrier to implementing an ASP.

Several comments highlighted important elements of our ASP. First, respondents noted the ASP was helpful in navigating the gray areas and complexity of clinical care of IC patients. These themes have been observed in prior surveys and underscore the importance of including IC patients in practice-changing studies evaluating appropriate spectrum and duration of antimicrobials. Comments suggest that IC ASPs can still guide practice and provide valuable education even in areas of clinical uncertainty.

Second, respondents noted the ASP is helpful for antimicrobial questions that do not rise to the level of requiring ID consultation or for determining when ID consult may be appropriate. These comments highlight the complementary roles of ASPs and ID consultation and suggest that availability of ASPs may lower the psychological barrier for asking questions about antimicrobials and infection syndromes. Lastly, the “in-person” component of our ASP was critical; we suspect this improved relationships, felt more collegial, facilitated discussion of nuances and gray areas, and therefore influenced willingness to change prescribing without negative feelings. In-person interactions may provide a “face” to ASP recommendations and signal that the ASP team is actively invested in the care of individual patients rather than functioning solely as a remote advisory service. This finding aligns with prior survey data suggesting a higher willingness to de-escalate antibiotics among patients with febrile neutropenia if it was paired with in-person ASP support.^13^

Strengths of this study include a high response rate (55%) spread between services and clinical roles. Limitations include that this was a single center study and it is unclear whether specific elements of our ASP were particularly important. Our program includes several structural features that may have influenced provider perceptions, including strong ID physician involvement, established stewardship culture, and a significant in-person component to ASP activities. These factors may not be present in all institutions and may influence the generalizability of our findings. In addition, we specifically implemented our ASP at our cancer center; it is unclear whether a similar ASP would be met with approval in non-cancer IC populations. We did not report impact on antimicrobial use, limiting conclusions about prescribing changes; however, nearly 80% of responses reported following ASP recommendations. Lastly, non-respondents of our survey may have different opinions than those who responded.

In conclusion, although there are limited studies of provider perceptions of ASP activities, our ASP consisting of in-person PAF, guidelines, education, and availability through a pager was viewed positively by providers. Fear of negative provider perceptions should not be a barrier to implementing IC ASPs at other institutions.

## Supporting information

10.1017/ash.2026.10756.sm001 Pallotta et al. supplementary material 1 Pallotta et al. supplementary material

10.1017/ash.2026.10756.sm002 Pallotta et al. supplementary material 2 Pallotta et al. supplementary material

## Supplementary material

The supplementary material for this article can be found at https://doi.org/10.1017/ash.2026.10756.
