# Supplementary material for: Provider perceptions of an antimicrobial stewardship program in immunocompromised patients at a cancer center
Source: Antimicrob Steward Healthc Epidemiol. 2026 Jul 3;6(1):e197. doi: 10.1017/ash.2026.10756 (PMC13343336; doi:10.1017/ash.2026.10756)
Supplement: Pallotta et al. supplementary material 1 — Pallotta et al. supplementary material [file S2732494X26107566sup001.docx]

Supplement Table 1: Demographics

| **Characteristic** | **No. (%) of Participants [n=65]** |
| --- | --- |
| *Gender* |  |
| Male | 29.2 |
| Female | 70.8 |
| *Occupational* *role* |  |
| Attending physician | 35.4 |
| Advanced practice clinician | 26.1 |
| Pharmacist | 38.5 |
| *Service** |  |
| Bone marrow transplant | 29.2 |
| Hematology | 50.8 |
| Medical oncology | 43.1 |
| *Length of clinical practice at Huntsman Cancer Institute* |  |
| <1 year | 9.2 |
| 1-5 years | 58.5 |
| 6-10 years | 16.9 |
| 11-16 years | 9.2 |
| >16 years | 6.2 |
