# Supplementary material for: Provider perceptions of an antimicrobial stewardship program in immunocompromised patients at a cancer center
Source: Antimicrob Steward Healthc Epidemiol. 2026 Jul 3;6(1):e197. doi: 10.1017/ash.2026.10756 (PMC13343336; doi:10.1017/ash.2026.10756)
Supplement: Pallotta et al. supplementary material 2 — Pallotta et al. supplementary material [file S2732494X26107566sup002.pdf]

Provider Attitudes Regarding ASP Survey

Consent Cover Letter

Provider attitudes regarding antimicrobial stewardship program activities

The purpose of this research study is to examine the experiences of clinicians and prescribers regarding their interactions with the antimicrobial stewardship team and activities. We are doing this study in order to identify the acceptability and feasibility of stewardship activities at the University of Utah, Huntsman Cancer Hospital, and Nielsen Rehabilitation Facility.

If you agree, you will participate in the below 5-10 minute survey about your experiences. Your participation is completely voluntary. You may choose not to answer a question or are free to discontinue participation in the survey at any time for any reason without penalty or loss of benefits.There are no known risks or benefits associated with participating in this study. The survey is anonymous and records will be kept confidential on a password-protected computer.

If you have any questions complaints or if you feel you have been harmed by this research please contact Hannah Imlay, hannah.imlay@hsc.utah.edu.

Contact the Institutional Review Board (IRB) if you have questions regarding your rights as a research participant. Also, contact the IRB if you have questions, complaints or concerns which you do not feel you can discuss with the investigator. The University of Utah IRB may be reached by phone at (801) 581-3655 or by e-mail at irb@hsc.utah.edu. Research Participant Advocate: You may also contact the Research Participant Advocate (RPA) by phone at (801) 581-3803 or by email at participant.advocate@hsc.utah.edu.

By participating in the survey, you are giving your consent to participate in this research. Thank you for your willingness to participate!

Please select your gender:

- ○ Female
- ○ Male
- ○ Nonbinary
- ○ Prefer not to answer

Please select your role on the care team:

- ○ Advance Practice Clinician (APC)
- ○ Attending Physician
- ○ Pharmacist

Please indicate the clinical services you currently work with (select all that apply):

- ☐ Medical oncology
- ☐ Bone marrow transplant (BMT)
- ☐ Hematology

Please indicate how long you have been in clinical practice at Huntsman Cancer Institute:

- ○ < 1 year
- ○ 1-5 years
- ○ 6-10 years
- ○ 11-16 years
- ○ >16 years

Please rank whether you agree or disagree with the following statements (strongly agree, agree, neutral, disagree, strongly disagree)

"Stewardship team" refers to physician/pharmacist who come to team rooms, review antibiotic use, and give feedback on non-ID consult patients

"Stewardship rounds" refers to the process of reviewing antibiotic use and giving feedback on non-ID consult patients

	Strongly disagree	Disagree	Neutral	Agree	Strongly agree
The stewardship team is respectful and collaborative.	○	○	○	○	○
It is easy to contact the stewardship pharmacist or physician when needed.	○	○	○	○	○
The stewardship team provides knowledge and education that helps improve my antibiotic use for future patients.	○	○	○	○	○
The stewardship team improves my clinical decision-making.	○	○	○	○	○
I trust the judgment of the stewardship team.	○	○	○	○	○
The stewardship team helps facilitate appropriate use of antimicrobials.	○	○	○	○	○
The stewardship team respects my clinical judgment.	○	○	○	○	○
The stewardship team OR stewardship rounds interferes with my clinical decision making.	○	○	○	○	○
The stewardship team OR stewardship improves my efficiency at work.	○	○	○	○	○
I would support the continuation of in-person stewardship rounds on our service.	○	○	○	○	○

On a scale of 1 (Completely Disagree) to 5 (Completely Agree), rate your agreement with the following statements:

"Stewardship team" refers to physician/pharmacist who come to team rooms, review antibiotic use, and give feedback on non-ID consult patients

"Stewardship rounds" refers to the process of reviewing antibiotic use and giving feedback on non-ID consult patients

	1. Completely Disagree	2. Disagree	3. Neither agree nor disagree	4. Agree	5. Completely Agree
The antimicrobial stewardship rounds meet my approval.	○	○	○	○	○
Antimicrobial stewardship rounds are appealing to me.	○	○	○	○	○
I like antimicrobial stewardship rounds.	○	○	○	○	○
I welcome antimicrobial stewardship rounds.	○	○	○	○	○

On a scale of often to not often (5-point scale) how often do the following happen:

	Never	Rarely	Sometimes	Often	Always
How often do you agree with the stewardship team's suggestions made during rounds	○	○	○	○	○
How often does your team follow the stewardship team's suggestions made during rounds	○	○	○	○	○

What has been most helpful about the stewardship rounds?

____

How could the process of stewardship rounds be improved?

____

General comments:

____
